# Sex differences in obesity incidence: 20‐year prospective cohort in South Africa

**DOI:** 10.1111/ijpo.12039

**Published:** 2015-05-19

**Authors:** E. A. Lundeen, S. A. Norris, L. S. Adair, L. M. Richter, A. D. Stein

**Affiliations:** ^1^ Nutrition and Health Sciences ProgramDivision of Biological and Biomedical SciencesLaney Graduate SchoolEmory UniversityAtlantaGAUSA; ^2^ Medical Research Council (MRC) Developmental Pathways for Health Research UnitUniversity of WitwatersrandJohannesburgSouth Africa; ^3^ Gillings School of Public HealthUniversity of North Carolina at Chapel HillChapel HillNCUSA; ^4^ Human Sciences Research CouncilDurbanSouth Africa; ^5^ Rollins School of Public HealthHubert Department of Global HealthEmory UniversityAtlantaGAUSA

**Keywords:** Body mass index, child growth, childhood overweight and obesity

## Abstract

**Background:**

Prospective data spanning childhood and adolescence are needed to better understand obesity incidence among children and to identify important periods for intervention.

**Objective:**

To describe gender differences in overweight and obesity from infancy to late adolescence in a South African cohort.

**Methods:**

We analysed body mass index at 1–2 years, 4–8 years, 11–12 years, 13–15 years and 16–18 years among 1172 participants in the South African Birth‐to‐Twenty cohort.

**Results:**

Among boys, overweight and obesity prevalence declined from age 1–2 years to 16–18 years. Among girls, overweight and obesity prevalence increased from 4–8 years to 16–18 years. Obesity incidence was highest from 4–8 years to 11–12 years in boys (6.8 cases per 1000 person‐years) and from 11–12 years to 13–15 years in girls (11.2 cases per 1000 person‐years). Among girls, obesity at 16–18 years was associated with overweight (odds ratio [OR] = 3.6; 95% confidence interval [CI] 1.8–7.2) or obesity (OR = 8.0; 95% CI 3.7–17.6) at 1–2 years and overweight (OR = 6.8; 95% CI 3.3–13.9) or obesity (OR = 42.3; 95% CI 15.0–118.8) at 4–8 years; for boys, obesity at 16–18 years was associated with overweight at 1–2 years (OR = 5.6; 95% CI 1.7–18.0) and obesity at 4–8 years (OR = 19.7; 95% CI 5.1–75.9).

**Conclusions:**

Among girls, overweight and obesity increased throughout childhood. Overweight and obesity were not widely prevalent among boys. Early childhood and post‐puberty may be important periods for intervention among girls.

## Introduction

The increasing prevalence of overweight and obesity is a growing concern in most regions of the world. Recent estimates have shown that the combined prevalence of overweight and obesity among adults globally has risen by 28% in the last three decades, with 37% of men and 38% of women having a body mass index (BMI) of 25 kg m^−2^ or greater 1. Obese adults have a greatly increased risk of chronic diseases such as diabetes and cardiovascular disease 2. This disease pathway may have its origins early in life, as childhood overweight and obesity have been shown to track into adulthood 3, 4, 5. Therefore, the increasing prevalence of overweight and obesity among children is a major public health concern 6.

From 2008 to 2013, the global prevalence of overweight and obesity among children 2–19 years increased by 47% 1. By 2013, the combined prevalence of overweight and obesity was 24% among boys and 23% among girls in high‐income countries and 13% among both boys and girls in low‐ and middle‐income countries 1. For many countries in Africa, childhood malnutrition is characterized primarily by undernutrition and this region has a lower prevalence of overweight and obesity than other regions of the world. However, over the past 20 years, South Africa has undergone dramatic and rapid social and economic transitions. Recent figures show that in sub‐Saharan Africa, South Africa has one of the highest prevalence rates of childhood (<20 years) overweight and obesity – 19% of boys and 26% of girls – rivalling that of many high‐income countries 1.

Understanding childhood obesity trends and important periods for intervention requires data not only on the prevalence, but also the incidence of obesity during key stages of childhood and adolescence. Few high‐income countries have population‐based data on the incidence of obesity throughout childhood and these data are essentially absent in low‐ and middle‐income country settings, where prevalence estimates are typically derived from cross‐sectional surveys and focus on children under 5 years. Longitudinal data spanning childhood and adolescence are needed. Such data are particularly sparse in sub‐Saharan Africa 7. We address this research gap by investigating the incidence of overweight and obesity among children in a birth cohort in Soweto, South Africa.

## Methods

We analysed data from the Birth‐to‐Twenty study, a birth cohort initiated in 1990 in Soweto, an urban township in South Africa. Detailed information on this cohort has been published elsewhere 8. The cohort enrolled 3273 pregnant women at gestational age 26–40 weeks, who were expected to deliver during a 6‐week period in early 1990. Participants were predominantly Black women with a low socioeconomic status. The study was designed to track the growth, health, well‐being and educational progress of their children, who have been studied prospectively. Weight at birth and weight and length/height at subsequent rounds were measured using standard procedures 9. Throughout the study, participants or their caregivers provided written informed consent and ethical approval was obtained from the University of the Witwatersrand Committee for Research on Human Subjects (approval ID #M010556).

Using weight, length and height measurements at each survey round, BMI values were calculated as weight (kg) divided by height squared (m^2^) and these values were then converted to z scores (BMIZ) using the World Health Organization (WHO) references 10, 11, 12. As not all children had BMI values for every year of measurement, to maximize data availability, we grouped survey rounds as follows: infant/toddler (1 year and 2 years), early childhood (4 years, 5 years and 8 years), early adolescence (11 years and 12 years), mid‐adolescence (13 years, 14 years and 15 years) and late adolescence (16 years, 17 years and 18 years). When more than one BMIZ value was available within a period, one was selected at random. We included in the analysis all participants who had a BMIZ for all five time periods (*n* = 1172). The large majority (88%) of participants were Black, with other represented groups including white (2%), Indian (1%) and coloured or mixed‐ancestral (9%) children.

We defined overweight and obesity for all five time periods using the WHO cut‐off points for children 5–19 years: overweight was defined as a BMIZ > 1.0 and ≤ 2.0 standard deviation (SD) from the reference median; obese was a BMIZ > 2.0 SD from the reference median 12. To enable comparisons with other studies, we also used definitions from the US Centers for Disease Control and Prevention (CDC) 13 and the International Obesity Task Force (IOTF) 14. We computed the prevalence of overweight and obesity at each time period. Incidence of obesity was calculated as the percentage of children who were not obese in the earlier time period who became obese by the subsequent time period. To account for the different durations of the intervals between periods, for each interval, we also computed the incidence density rate as the number of incident cases per 1000 person‐years, where person‐years was computed by summing the number of years of follow‐up contributed by each child at risk of becoming obese during that period.

We computed a measure of the persistence of obesity by calculating the proportion of children who became obese at any point during the 1–2 years to 13–15 years periods that remained obese in the 16–18 years period. We used logistic regression to model the risk of being obese at 16–18 years based on status at the 1–2 years or 4–8 years periods, with the referent category being those who were classified as normal BMI (not overweight or obese) at that time period. We also calculated the proportion of children who were obese in the earlier time period who became non‐obese by the subsequent time period.

A sensitivity analysis was performed using two methods to determine whether there was a systematic difference between the cohort of children who had data at all five time periods and those who did not. We calculated overweight and obesity prevalence figures using all available cross‐sectional data at each of the time points and compared them with the prevalence figures for children who had complete longitudinal data. Additionally, we compared overweight and obesity prevalence figures for children with complete longitudinal data vs. those who were not included in the analysis. We used STATA 10.0 (StataCorp, College Station, TX, USA) statistical program for all data analyses.

## Results

For both boys and girls, mean BMI was around 16.8 kg m^−2^ at 1–2 years, decreased slightly by 4–8 years and then increased steadily through 16–18 years (Table 1). At 16–18 years, mean BMI was 22.8 kg m^−2^ in girls and 20.3 kg m^−2^ in boys. Using the WHO reference, mean BMIZ at 1–2 years was 0.3 and 0.4 in boys and girls, respectively, and decreased to −0.2 in boys and −0.03 in girls at 11–12 years. Thereafter, mean BMIZ continued to decline in boys, dropping to −0.6 at 16–18 years, but rose among girls, reaching 0.3 at 16–18 years.

**Table 1:** Measures of overweight and obesity among South African boys (*n* = 566) and girls (*n* = 606) from 1–2 years to 16–18 years,* Birth‐to‐Twenty Cohort

	1–2 years^†††^	4–8 years	11–12 years	13–15 years	16–18 years
BMI^†§^					
Boys	16.9 (16.7, 17.0)	15.8 (15.6, 15.9)	17.8 (17.5, 18.0)	19.2 (18.9, 19.5)	20.3 (20.0, 20.6)
Girls	16.7 (16.5, 16.8)	15.7 (15.6, 15.8)	18.8 (18.5, 19.2)	21.3 (21.0, 21.7)	22.8 (22.5, 23.2)
BMIZ^‡§^					
Boys	0.3 (0.2, 0.4)	0.2 (0.1, 0.2)	−0.2 (−0.3, −0.1)	−0.4 (−0.5, −0.3)	−0.6 (−0.7, −0.5)
Girls	0.4 (0.3, 0.5)	0.1 (0.0, 0.2)	−0.03 (−0.1, 0.1)	0.2 (0.1, 0.3)	0.3 (0.2, 0.4)
Obesity^¶^					
WHO definition**					
Boys	8.8 (6.5, 11.2)	3.0 (1.6, 4.4)	6.0 (4.0, 8.0)	4.4 (2.7, 6.1)	2.5 (1.2, 3.8)
Girls	8.1 (5.9, 10.3)	3.1 (1.7, 4.5)	6.4 (4.5, 8.4)	7.3 (5.2, 9.3)	7.9 (5.8, 10.1)
IOTF definition^††^					
Boys	4.8 (3.0, 6.5)	0.9 (0.1, 1.7)	3.2 (1.7, 4.6)	3.2 (1.7, 4.6)	1.9 (0.8, 3.1)
Girls	4.8 (3.1, 6.5)	2.3 (1.1, 3.5)	5.1 (3.4, 6.9)	6.1 (4.2, 8.0)	7.4 (5.3, 9.5)
CDC definition^‡‡^					
Boys	13.8 (10.9, 16.6)	6.0 (4.0, 8.0)	9.0 (6.6, 11.4)	6.2 (4.2, 8.2)	3.5 (2.0, 5.1)
Girls	12.4 (9.7, 15.0)	5.3 (3.5, 7.1)	9.9 (7.5, 12.3)	12.5 (9.9, 15.2)	12.7 (10.0, 15.4)
Overweight^¶§§^					
WHO definition^¶¶^					
Boys	19.1 (15.8, 22.3)	16.4 (13.4, 19.5)	9.9 (7.4, 12.4)	7.8 (5.6, 10.0)	5.7 (3.7, 7.6)
Girls	19.0 (15.8, 22.1)	12.2 (9.6, 14.8)	14.7 (11.9, 17.5)	17.8 (14.8, 20.9)	19.1 (16.0, 22.3)
IOTF definition^††^					
Boys	12.7 (10.0, 15.5)	6.5 (4.5, 8.6)	9.0 (6.6, 11.4)	7.4 (5.3, 9.6)	6.9 (4.8, 9.0)
Girls	14.5 (11.7, 17.3)	7.6 (5.5, 9.7)	12.5 (9.9, 15.2)	16.0 (13.1, 18.9)	19.0 (15.8, 22.1)
CDC definition***					
Boys	12.4 (9.6, 15.1)	12.4 (9.6, 15.1)	6.7 (4.6, 8.8)	5.7 (3.7, 7.6)	4.6 (2.9, 6.3)
Girls	13.5 (10.8, 16.3)	9.2 (6.9, 11.6)	11.1 (8.6, 13.6)	12.0 (9.4, 14.6)	13.7 (11.0, 16.4)

*Infant/toddler – 1 year or 2 years; early childhood – 4 years, 5 years or 8 years; early adolescence – 11 years or 12 years; mid‐adolescence – 13 years, 14 years and 15 years; late adolescence – 16 years, 17 years and 18 years. ^†^Body mass index. ^‡^Body mass index z score. ^§^Mean (95% confidence interval). ^¶^% (95% confidence interval). **World Health Organization (WHO) defines obesity as BMI z score > 2.0 standard deviation (SD) from the reference median. ^††^International Obesity Task Force (IOTF) defines obesity and overweight using age‐ and sex‐specific cut‐off points for children 2–18 years, which were based on models that adapted the adult cut‐off points for overweight (25 ≤ BMI < 30) and obese (BMI ≥ 30). ^‡‡^Centers for Disease Control (CDC) define obesity as BMI ≥ 95th percentile based on the reference population. ^§§^Overweight, but not obese. ^¶¶^WHO defines overweight as BMIZ > 1.0 and ≤ 2.0 SD from the reference median. ***CDC define overweight as BMI ≥ the 85th and <95th percentile based on the reference population. ^†††^The IOTF and CDC definitions for overweight/obesity are for children 2–18 years and 2–19 years, respectively. IOTF and CDC prevalence figures for the infancy/toddlerhood period should be interpreted with caution, as this period contains BMI measurements at 1 year and 2 years of age. IOTF and CDC cut‐off points for 2 years were applied to the measurements at 1 year.

Among boys, the prevalence of overweight was highest through 4–8 years and declined thereafter, while obesity was highest at ages 1–2 years and 11–12 years, but decreased to <3% by 16–18 years. Among girls, the prevalence of overweight increased steadily after 4–8 years, reaching 19% by 16–18 years. The prevalence of obesity in girls also continued to rise after 4–8 years, reaching 8% by 16–18 years. While the absolute numbers differ, similar patterns were seen when the IOTF and CDC definitions of overweight and obesity were used.

For both boys and girls, the period incidence of obesity was highest from 4–8 years to 11–12 years (Table 2). This pattern held true for boys when the incidence density rate was compared. However, among girls, the incidence density rate was highest in the periods from 11–12 years to 13–15 years and from 13–15 years to 16–18 years. In some periods, e.g. from 1–2 years to 4–8 years, incident obesity is observed, however, there was an overall decreasing prevalence of obesity due to the majority of transition in that period being reversion from obese to non‐obese (Table 2).

**Table 2:** Incidence of obesity among South African boys (*n* = 566) and girls (*n* = 606) from 1–2 years to 16–18 years,* Birth‐to‐Twenty Cohort

	1–2 years to 4–8 years	4–8 years to 11–12 years	11–12 years to 13–15 years	13–15 years to 16–18 years
Obesity^†^ incidence				
Period incidence^‡^				
Boys	2.1 (0.9, 3.4)	4.4 (2.7, 6.1)	0.9 (0.1, 1.8)	0.4 (−0.1, 0.9)
Girls	2.0 (0.8, 3.1)	4.9 (3.2, 6.7)	2.8 (1.5, 4.2)	3.0 (1.6, 4.4)
Incidence density rate^§^				
Boys	5.1 (2.8, 9.1)	6.8 (4.6, 10.2)	3.7 (1.5, 8.8)	1.3 (0.3, 5.3)
Girls	5.0 (2.8, 9.0)	7.4 (5.2, 10.7)	11.2 (6.9, 18.3)	11.0 (6.8, 17.7)
Reversion incidence				
Period incidence^¶^				
Boys	88.0 (78.7, 97.3)	41.2 (15.1, 67.3)	41.2 (23.7, 58.6)	52.0 (31.0, 73.0)
Girls	83.7 (72.9, 94.4)	47.4 (22.6, 72.1)	28.2 (13.4, 43.0)	29.5 (15.5, 43.6)

*Infant/toddler – 1 year or 2 years; early childhood – 4 years, 5 years or 8 years; early adolescence – 11 years or 12 years; mid‐adolescence – 13 years, 14 years and 15 years; late adolescence – 16 years, 17 years and 18 years. ^†^World Health Organization defines obesity as body mass index z score > 2.0 standard deviation from the reference median. ^‡^Incident obesity cases during the period among those who were at risk for obesity at the beginning of the period; % (95% CI). ^§^Number of incident obesity cases during the period per 1000 person‐years. ^¶^Incident cases of reverting from obese to non‐obese during the period among those who were obese at the beginning of the period; % (95% CI)

Girls who were overweight at 1–2 years had 3.6 times increased odds of being obese from 16 to 18 years (*P* < 0.001), while those who were obese had 8.0 times greater odds (*P* < 0.001) (Table 3). Girls who were overweight at 4–8 years had 6.8 times increased odds of being obese from 16 to 18 years (*P* < 0.001), while those who were obese had 42.3 times greater odds (*P* < 0.001). Among females who became obese from the 1–2 years to 13–15 years periods (*n* = 97), obesity was persistent, or present during 16–18 years, in 36.1% (data not shown).

**Table 3:** Modelling risk of obesity from 16–18 years based on body mass index status at 1–2 years and 4–8 years among South African boys (*n* = 566) and girls (*n* = 606), Birth‐to‐Twenty Cohort^†^

	Odds Ratio	95% confidence interval
Girls		
Infancy/toddlerhood (1–2 years)		
Overweight	3.6***	1.8, 7.2
Obese	8.0***	3.7, 17.6
Early childhood (4–8 years)		
Overweight	6.8***	3.3, 13.9
Obese	42.3***	15.0, 118.8
Boys		
Infancy/toddlerhood (1–2 years)		
Overweight	5.6**	1.7, 18.0
Obese	3.4	0.6, 17.8
Early childhood (4–8 years)		
Overweight	2.1	0.5, 8.4
Obese	19.7***	5.1, 75.9

^†^Models the risk of being obese at late adolescence (16 years, 17 years and 18 years) based on status at the infant/toddler (1 year or 2 years) or early childhood periods (4 years, 5 years or 8 years), with the referent category being those who were classified as normal body mass index (not overweight or obese) at that time period. *P‐value <0.05; **P‐value <0.01; ***P‐value <0.001.

Boys who were overweight at 1–2 years had 5.6 times increased odds of being obese from 16 to 18 years (*P* < 0.01), while the increased odds conferred by obesity in this period were not significant (odds ratio [OR] = 3.4; *P* = 0.15) (Table 3). Boys who were obese at 4–8 years had 19.7 times increased odds of being obese from 16 to 18 years (*P* < 0.001), while overweight in this period did not result in significantly increased odds (OR = 2.1; *P* = 0.28). Among boys who became obese from the 1–2 years to 13–15 years periods (*n* = 83), obesity was persistent, or present during 16–18 years, in 16.9% (data not shown).

## Discussion

A key finding of our study is that in this cohort of South African children residing in a relatively poor but highly transitioned urban setting, boys and girls have very different patterns of incidence of overweight and obesity. A major strength of our analysis is that it was based on longitudinal data that spanned childhood. This is particularly important in low‐ and middle‐income countries, especially African countries, where such data are sparse.

The prevalence of overweight and obesity among boys does not represent a significant public health concern. In boys, the combined prevalence of overweight and obesity (BMIZ > 1.0) steadily declined and by late adolescence, it was less than the 16% that would be expected to be >1 SD from the reference (based on the percentage of observations in the tails of a normal distribution). However, among girls, the prevalence of combined overweight and obesity continued to rise, reaching 11 percentage points greater than the 16% expected for a normal distribution by late adolescence.

We found that among girls, the highest obesity incidence density rates occurred from the 11–12 years to 13–15 years and 13–15 years to 16–18 years periods. These periods typically follow the onset of puberty, a time of rapid growth and development, as well as sexual dimorphism, with females acquiring more fat mass than males, who gain greater amounts of muscle and skeletal mass. The average age of menarche for girls in our sample was 12.6 years of age, with 36% of girls having experienced menarche by the 11–12 years period, 90% by the 13–15 years period and 100% by the 16–18 years period. Among girls, there was also evidence of development of obesity early in life and obesity tracking into adulthood. There was an appreciable degree of persistence of obesity following its onset and both overweight and obese girls in the 1–2 years and 4–8 years periods had greatly increased odds of being obese by 16–18 years.

The prevalence of obesity among both boys and girls in this South African cohort is lower than their counterparts in high‐income countries. In a contemporary cohort of US children, Cunningham *et al* found a much higher prevalence of obesity: around 12–13% at age 5–7 years, 22% at age 11 years and 21% at age 14 years 15. That study did not extend to late adolescence. Obesity prevalence in our sample is lower than that of 12–19‐year‐old African–American boys (21%) and girls (23%) 16.

Our estimates for overweight and obesity by 16–18 years were similar to those found in the ethnically comparable 2012 South Africa National Health and Nutrition Examination Survey: 8.0% obese and 19.3% overweight among females 15–17 years; 1.5% obese and 7.3% overweight among males 15–17 years 17 and the prevalence of overweight and obesity among South African high‐school age adolescents is rising 18. While the overweight and obesity prevalence figures for South African children, particularly boys, are still lower than those of higher‐income countries, the latest adult obesity prevalence figures for South Africa are comparable with those seen in higher‐income countries and are the highest among all countries in Africa 1. Among South African men > 20 years of age, 39% are overweight or obese, as are 69% of women 1. Thus, overweight and obesity continue to accrue in the adult years and the majority of adult obesity is occurring after late adolescence 17. These statistics underscore the urgency to find effective interventions to prevent the development of obesity in the adult years, in addition to preventing its early onset in childhood.

We chose to apply to all time periods the WHO overweight and obesity thresholds for children 5–19 years. This allowed for consistency in definitions across the study, but may have overestimated the prevalence of overweight and obesity in the age 1–2 years period compared with using the WHO thresholds for children 0–5 years. Additionally, the analytical sample was limited to the 1172 children who had a BMI value for each of the five time periods. However, we found that the overweight and obesity prevalence figures based on all available cross‐sectional data at each of the time points did not differ appreciably from the figures based on those who had complete longitudinal data (Table S1) and there were no statistically significant differences in any of the time periods between the overweight and obesity prevalence figures for children with complete longitudinal data vs. those who were not included in the analysis (Table S2). Therefore, selection bias is unlikely. The cohort is representative of the population of Soweto and is overwhelmingly Black, with too few non‐Black participants to permit meaningful stratified analysis. Restriction of the analysis to the Black participants resulted in estimates very close to those presented.

Our results suggest the need for further research into gender differences in overweight and obesity. Future studies should explore dietary and lifestyle factors that may contribute to the differences found between South African boys and girls. Feeley *et al* assessed the relationship between dietary habits and BMIZ and fat mass in the Birth‐to‐Twenty cohort, finding an association between soft drink consumption and increased BMIZ and fat mass, but only in boys 19. Kruger *et al* found that South African girls had lower levels of physical activity than boys and, in both genders, low activity levels were associated with overweight and obesity 20. Another important area for future research is to explore gender differences in the interpretation of BMI to characterize overweight and obesity.

## Conclusion

Our findings have important implications not only for South Africa, but for other African countries that are rapidly transitioning. Understanding periods of high risk for the development of childhood obesity will enable programme planners and policymakers in low‐ and middle‐income countries to more appropriately target interventions. Our findings are important for addressing obesity risk across the life course. By late adolescence, overweight and obesity were not widely prevalent among boys in this South African cohort, whereas overweight and obesity among girls increased throughout childhood and adolescence. The years following puberty appear to be a high risk period for the development of obesity in girls and future research should further explore ways in which puberty timing and duration impact this risk. The early establishment of tracking of overweight and obesity suggests the need and potential for early intervention. Finding effective and appropriately timed interventions to promote healthy nutrition and weight among female African children is critical.

## Author contributions

LMR and SAN were involved in the design and implementation of the Birth‐to‐Twenty study in South Africa. EAL, SAN and ADS oversaw the initial design of the analysis, wrote the paper and had primary responsibility for the final content. EAL analysed the data. All authors interpreted the data, helped prepare the manuscript and approved the final version. EAL and ADS had full access to the data in the study and final responsibility for the decision to submit for publication.

## Funding

This work was supported by the Laney Graduate School, Emory University. The Birth‐to‐Twenty Study receives support from Wellcome Trust (UK), South African Medical Research Council, University of the Witwatersrand, Johannesburg, the Human Sciences Research Council and the UK MRC/DfID Africa Research Leader Scheme.

## Conflict of Interest Statement

No conflict of interest was declared.

## Supporting information


**Table S1.** Measures of overweight and obesity among South African boys and girls from 1–2 years to 16–18 years,^1^ Birth‐to‐Twenty Cohort (Sensitivity analysis: presenting figures based on cross‐sectional, or all available, data at each time period).
**Table S2.** Measures of overweight and obesity among South African boys and girls from 1–2 years to 16–18 years,^1^ Birth‐to‐Twenty Cohort (Sensitivity analysis: presenting figures based on individuals who were lost to follow‐up and not in the main analysis).

## References

[1] Ng M , Fleming T , Robinson M , *et al* Global, regional, and national prevalence of overweight and obesity in children and adults during 1980–2013: a systematic analysis for the Global Burden of Disease Study 2013. Lancet 2014; 384: 766–781. 24880830 10.1016/S0140-6736(14)60460-8 PMC4624264

[2] Guh DP , Zhang W , Bansback N , Amarsi Z , Birmingham CL , Anis AH . The incidence of co‐morbidities related to obesity and overweight: a systematic review and meta‐analysis. BMC Public Health 2009; 9: 1–20. 19320986 10.1186/1471-2458-9-88 PMC2667420

[3] Dietz WH . Critical periods in childhood for the development of obesity. Am J Clin Nutr 1994; 59: 955–959. 8172099 10.1093/ajcn/59.5.955

[4] Serdula MK , Ivery D , Coates RJ , Freedman DS , Williamson DF , Byers T . Do obese children become obese adults? A review of the literature. Prev Med 1993; 22: 167–177. 8483856 10.1006/pmed.1993.1014

[5] Singh AS , Mulder C , Twisk JWR , van Mechelen W , Chinapaw MJM . Tracking of childhood overweight into adulthood: a systematic review of the literature. Obes Rev 2008; 9: 474–488. 18331423 10.1111/j.1467-789X.2008.00475.x

[6] Black RE , Victora CG , Walker SP , *et al* Maternal and child undernutrition and overweight in low‐income and middle‐income countries. Lancet 2013; 382: 427–451. 23746772 10.1016/S0140-6736(13)60937-X

[7] Lobstein T , Baur L , Uauy R . Obesity in children and young people: a crisis in public health. Obes Rev 2004; 5(Suppl. 1): 4–85. 15096099 10.1111/j.1467-789X.2004.00133.x

[8] Richter L , Norris S , Pettifor J , Yach D , Cameron N . Cohort profile: Mandela's children: the 1990 Birth to Twenty study in South Africa. Int J Epidemiol 2007; 36: 504–511. 17355979 10.1093/ije/dym016 PMC2702039

[9] Cameron N . The Measurement of Human Growth. Croom‐Helm: London, UK, 1984.

[10] WHO Multicentre Growth Reference Study Group . WHO Child Growth Standards based on length/height, weight and age. Acta Paediatr Suppl 2006; 450: 76–85. 16817681 10.1111/j.1651-2227.2006.tb02378.x

[11] World Health Organization. WHO Multicentre Growth Reference Study Group . WHO Child Growth Standards: Length/Height‐for‐Age, Weight‐for‐Age, Weight‐for‐Length, Weight‐for‐Height and Body Mass Index‐for‐Age: Methods and Development. World Health Organization: Geneva, 2006.

[12] de Onis M , Onyango A , Borghi E . Development of a WHO growth reference for school‐aged children and adolescents. Bull World Health Organ 2007; 85: 660–667. 18026621 10.2471/BLT.07.043497 PMC2636412

[13] Kuczmarski R , Ogden C , Grummer‐Strawn L . CDC Growth Charts: United States. National Center for Health Statistics: Hyattsville, MD, 2000.

[14] Cole TJ , Bellizzi MC , Flegal KM , Dietz WH . Establishing a standard definition for child overweight and obesity worldwide: international survey. BMJ 2000; 320: 1240–1245. 10797032 10.1136/bmj.320.7244.1240 PMC27365

[15] Cunningham SA , Kramer MR , Narayan KMV . Incidence of childhood obesity in the United States. N Engl J Med 2014; 370: 1660–1661. 24758623 10.1056/NEJMc1402397 PMC7857670

[16] Ogden CL , Carroll MD , Kit BK , Flegal KM . Prevalence of childhood and adult obesity in the United States, 2011–2012. JAMA 2014; 311: 806–814. 24570244 10.1001/jama.2014.732 PMC4770258

[17] Shisana O , Labadarios D , Rehle T , *et al* South African National Health and Nutrition Examination Survey (SANHANES‐1). HSRC Press: Cape Town, 2014.

[18] Reddy SP , Resnicow K , James S , *et al* Rapid increases in overweight and obesity among South African adolescents: comparison of data from the South African national youth risk behaviour survey in 2002 and 2008. Am J Public Health 2012; 102: 262–268. 21940919 10.2105/AJPH.2011.300222 PMC3483977

[19] Feeley AB , Musenge E , Pettifor JM , Norris SA . Investigation into longitudinal dietary behaviours and household socio‐economic indicators and their association with BMI Z‐score and fat mass in South African adolescents: the Birth to Twenty (Bt20) cohort. Public Health Nutr 2012; 16: 693–703. 22801035 10.1017/S1368980012003308 PMC10271871

[20] Kruger R , Kruger H , MacIntyre U . The determinants of overweight and obesity among 10‐ to 15‐ year‐old schoolchildren in the North West Province, South Africa – the THUSA BANA (Transition and Health during Urbanisation of South Africans; BANA, children) study. Public Health Nutr 2006; 9: 351–358. 16684387 10.1079/phn2006849

