# Supplementary material for: Sex differences in obesity incidence: 20‐year prospective cohort in South Africa
Source: Pediatr Obes. 2015 May 19;11(1):75–80. doi: 10.1111/ijpo.12039 (PMC4832364; doi:10.1111/ijpo.12039)
Supplement: Supplementary file 1 — Table S1. Measures of overweight and obesity among South African boys and girls from 1–2 years to 16–18 years,1 Birth‐to‐Twenty Cohort (Sensitivity analysis: presenting figures based on cross‐sectional, or all available, data at each time period). Table S2. Measures of overweight and obesity among South African boys and girls from 1–2 years to 16–18 years,1 Birth‐to‐Twenty Cohort (Sensitivity analysis: presenting figures based on individuals who were lost to follow‐up and not in the main analysis). [file IJPO-11-75-s001.pdf]

Supporting Information: Appendices 1 and 2

Sex differences in obesity incidence: 20-year prospective cohort in South Africa

Elizabeth A Lundeen,[1, 2] Shane A Norris,[2] Linda S Adair,[3, 2] Linda M Richter,[4, 2] Aryeh D Stein[5, 2]

1 Nutrition and Health Sciences Program, Division of Biological and Biomedical Sciences, Laney Graduate School, Emory University, Atlanta, USA; 2 MRC Developmental Pathways for Health Research Unit, University of Witwatersrand, Johannesburg, South Africa; 3 Gillings School of Public Health, University of North Carolina - Chapel Hill, Chapel Hill, USA; 4 Human Sciences Research Council, Durban, South Africa; 5 Rollins School of Public Health, Hubert Department of Global Health, Emory University, Atlanta, USA

Corresponding author:
Aryeh D. Stein
Professor
Hubert Department of Global Health and Department of Epidemiology
Rollins School of Public Health, Emory University
Mail: Mailstop 1518-002-7BB, Emory University, Atlanta GA 30322, USA
Tel: (+1) 404 727 4255; Fax: (+1) 404 727 1278; email: aryeh.stein@emory.edu

Appendix 1. Measures of overweight and obesity among South African boys and girls from 1-2y to 16-18y[1], Birth-to-Twenty Cohort (Sensitivity analysis: presenting figures based on cross-sectional, or all available, data at each time period)

	1-2y[9] (n=2,163)	4-8y (n=2,162)	11-12y (n=1,839)	13-15y (n=2,192)	16-18y (n=2,113)
**Obesity[2]**					
WHO definition[3]					
Boys	9.4 (7.6, 11.1)	3.4 (2.3, 4.5)	5.5 (4.0, 7.0)	4.5 (3.2, 5.7)	3.0 (1.9, 4.0)
Girls	9.1 (7.5, 10.8)	2.9 (1.9, 3.9)	6.7 (5.2, 8.3)	7.9 (6.3, 9.4)	8.7 (7.1, 10.4)
IOTF definition[4]					
Boys	5.0 (3.6, 6.3)	1.5 (0.8, 2.3)	3.2 (2.0, 4.4)	3.6 (2.5, 4.8)	2.6 (1.6, 3.5)
Girls	5.7 (4.4, 7.1)	2.0 (1.1, 2.8)	5.3 (3.9, 6.7)	6.6 (5.1, 8.0)	8.1 (6.5, 9.7)
CDC definition[5]					
Boys	15.0 (12.8, 17.1)	6.9 (5.4, 8.5)	8.7 (6.8, 10.5)	6.2 (4.7, 7.7)	4.1 (2.9, 5.4)
Girls	14.0 (12.0, 16.0)	5.5 (4.2, 6.8)	10.6 (8.6, 12.5)	12.4 (10.5, 14.3)	13.0 (11.0, 15.0)
**Overweight[2, 6]**					
WHO definition[7]					
Boys	18.8 (16.4, 21.2)	15.7 (13.5, 17.9)	9.8 (7.8, 11.8)	7.1 (5.6, 8.7)	5.3 (3.9, 6.7)
Girls	18.7 (16.5, 21.0)	12.3 (10.3, 14.2)	15.1 (12.8, 17.3)	15.8 (13.7, 18.0)	17.7 (15.4, 19.9)
IOTF definition[4]					
Boys	14.6 (12.5, 16.7)	6.4 (4.9, 7.9)	8.2 (6.4, 10.0)	6.8 (5.2, 8.3)	6.1 (4.6, 7.6)
Girls	15.6 (13.5, 17.7)	8.1 (6.5, 9.7)	13.2 (11.0, 15.3)	14.6 (12.6, 16.7)	17.7 (15.4, 19.9)
CDC definition[8]					
Boys	11.6 (9.6, 13.5)	11.3 (9.4, 13.2)	6.5 (4.9, 8.1)	5.1 (3.8, 6.5)	4.1 (2.9, 5.4)
Girls	12.7 (10.8, 14.7)	9.1 (7.4, 10.7)	10.9 (8.9, 12.9)	10.8 (9.0, 12.6)	12.7 (10.7, 14.6)

1 Infant/toddler – 1y or 2y; early childhood – 4y, 5y, or 8y; early adolescence – 11y or 12y; mid-adolescence – 13y, 14y, 15y; late adolescence – 16y, 17y, 18y
2 % (95% confidence interval)
3 World Health Organization (WHO) defines obesity as BMI z score > 2.0 SD from the reference median
4 International Obesity Task Force (IOTF) defines obesity and overweight using age- and sex-specific cut-off points for children 2-18 y, which were based on models that adapted the adult cut-off points for overweight (25≤BMI<30) and obese (BMI≥30).
5 Centers for Disease Control (CDC) define obesity as BMI ≥ 95th percentile based on the reference population
6 Overweight, but not obese
7 World Health Organization defines overweight as BMI z score > 1.0 and ≤ 2.0 SD from the reference median
8 Centers for Disease Control define overweight as BMI ≥ the 85th and < 95th percentile based on the reference population
9 The IOTF and CDC definitions for overweight/obesity are for children 2-18 years and 2-19 years, respectively. IOTF and CDC prevalence figures for the infancy/toddlerhood period should be interpreted with caution, as this period contains BMI measurements at 1y and 2y of age. IOTF and CDC cut-off points for 2y were applied to the measurements at 1y.

Appendix 2. Measures of overweight and obesity among South African boys and girls from 1-2y to 16-18y[1], Birth-to-Twenty Cohort (Sensitivity analysis: presenting figures based on individuals who were lost-to-follow-up and not in the main analysis)

	1-2y[9] (n=991)	4-8y (n=990)	11-12y (n=667)	13-15y (n=1,020)	16-18y (n=941)
**Obesity[2]**					
WHO definition[3]					
Boys	10.0 (7.3, 12.6)	3.8 (2.1, 5.6)	4.5 (2.2, 6.8)	4.6 (2.7, 6.4)	3.6 (1.8, 5.3)
Girls	10.4 (7.8, 13.1)	2.7 (1.3, 4.1)	7.3 (4.6, 10.0)	8.6 (6.2, 10.9)	9.8 (7.1, 12.4)
IOTF definition[4]					
Boys	5.2 (3.2, 7.2)	2.3 (1.0, 3.7)	3.2 (1.2, 5.2)	4.1 (2.4, 5.9)	3.3 (1.7, 5.0)
Girls	6.9 (4.7, 9.1)	1.5 (0.5, 2.6)	5.6 (3.2, 8.0)	7.1 (4.9, 9.3)	8.9 (6.4, 11.5)
CDC definition[5]					
Boys	16.4 (13.1, 19.7)	8.1 (5.6, 10.5)	8.1 (5.0, 11.1)	6.2 (4.1, 8.4)	4.9 (2.9, 6.9)
Girls	15.9 (12.7, 19.1)	5.8 (3.8, 7.8)	11.8 (8.4, 15.1)	12.3 (9.5, 15.1)	13.4 (10.4, 16.4)
**Overweight[2, 6]**					
WHO definition[7]					
Boys	18.5 (15.0, 21.9)	14.9 (11.6, 18.1)	9.7 (6.4, 13.0)	6.4 (4.2, 8.6)	4.9 (2.9, 6.9)
Girls	18.5 (15.1, 21.8)	12.3 (9.5, 15.2)	15.7 (11.9, 19.5)	13.6 (10.7, 16.5)	15.9 (12.6, 19.1)
IOTF definition[4]					
Boys	16.8 (13.5, 20.2)	6.2 (4.0, 8.3)	6.8 (4.0, 9.6)	6.0 (3.9, 8.1)	5.1 (3.1, 7.2)
Girls	16.9 (13.6, 20.2)	8.7 (6.2, 11.1)	14.3 (10.6, 17.9)	13.0 (10.2, 15.9)	16.1 (12.8, 19.3)
CDC definition[8]					
Boys	10.6 (7.8, 13.3)	10.0 (7.3, 12.7)	6.1 (3.4, 8.8)	4.6 (2.7, 6.4)	3.6 (1.8, 5.3)
Girls	11.8 (9.0, 14.6)	8.9 (6.4, 11.3)	10.6 (7.4, 13.9)	9.3 (6.8, 11.8)	11.4 (8.6, 14.2)

1 Infant/toddler – 1y or 2y; early childhood – 4y, 5y, or 8y; early adolescence – 11y or 12y; mid-adolescence – 13y, 14y, 15y; late adolescence – 16y, 17y, 18y
2 % (95% confidence interval)
3 World Health Organization (WHO) defines obesity as BMI z score > 2.0 SD from the reference median
4 International Obesity Task Force (IOTF) defines obesity and overweight using age- and sex-specific cut-off points for children 2-18 y, which were based on models that adapted the adult cut-off points for overweight (25≤BMI<30) and obese (BMI≥30).
5 Centers for Disease Control (CDC) define obesity as BMI ≥ 95th percentile based on the reference population
6 Overweight, but not obese
7 World Health Organization defines overweight as BMI z score > 1.0 and ≤ 2.0 SD from the reference median
8 Centers for Disease Control define overweight as BMI ≥ the 85th and < 95th percentile based on the reference population
9 The IOTF and CDC definitions for overweight/obesity are for children 2-18 years and 2-19 years, respectively. IOTF and CDC prevalence figures for the infancy/toddlerhood period should be interpreted with caution, as this period contains BMI measurements at 1y and 2y of age. IOTF and CDC cut-off points for 2y were applied to the measurements at 1y.
